# Dietary patterns and their association with cardiovascular risk factors in Ethiopia: A community-based cross-sectional study

**DOI:** 10.3389/fnut.2023.1074296

**Published:** 2023-03-23

**Authors:** Wondimagegn Paulos Kumma, Eskindir Loha

**Affiliations:** ^1^School of Public Health, Hawassa University, Hawassa, Ethiopia; ^2^Centre for International Health, University of Bergen, Bergen, Norway; ^3^School of Public Health, Wolaita Sodo University, Wolaita Sodo, Ethiopia; ^4^Chr. Michelsen Institute, Bergen, Norway

**Keywords:** dietary patterns, coexistence, cardiovascular disease risk factors, Wolaita, southern Ethiopia

## Abstract

**Purpose:**

To identify the dietary patterns and their association with cardiovascular risk factors among adult people in urban and rural areas of Wolaita, southern Ethiopia.

**Methods:**

A total of 2,483 participants aged 25–64 years were selected using a three-stage random sampling. Data for this study were collected using structured questionnaires, the previous 24-h dietary intake assessment, anthropometric, blood pressure, and biochemical measurements. We used factor analysis to identify dietary patterns. Factors associated with dietary patterns were analyzed using multiple linear regression models. The adjusted regression coefficients with their 95% CI were used to ascertain the association.

**Result:**

We identified three major dietary patterns that explained 51% of the variance in food consumption. The *western dietary pattern* was characterized by the consumption of meat/organ meat, biscuits/sweets, chicken stew, pasta-macaroni recipes, butter, white wheat bread, egg recipe, and Ethiopian dish *shiro-wet*, and was positively associated with urban residence, obesity, hypertension, blood glucose, and total cholesterol levels. Adherence to the consumption of tubers, whole-grain maize products, coffee leaves-and-herbs beverage, legumes, and sweet potatoes featured the *traditional dietary pattern*. The *traditional dietary pattern* showed a positive relationship with rural residence, physical activity, and obesity, and it had a negative relationship with hypertension. The *healthy dietary pattern* was characterized by the intake of green leafy vegetables, green pepper, and whole-grain maize products, and negatively related to obesity, and hypertension, while positively related to urban residence.

**Conclusion:**

The coexistence of *western, traditional, and healthy dietary patterns* in the present study indicates the transition to a new dietary pattern in the study area. All dietary patterns were associated with one or more cardiovascular risk factors, but the western dietary pattern was associated with most of these, while the traditional diet showed fewer such associations. Therefore, it might be useful to promote *healthy and traditional dietary patterns* along with physical activity. Interventions related to the current findings, if initiated early in life, may benefit the public in preventing cardiovascular risk factors such as obesity, hypertension, and type 2-diabetes.

## Introduction

The global average for dietary quality is low with the Alternative Healthy Eating Index (AHEI) ranging from 0 to 100, where 100 represent the healthiest diet. Among children and adults in 2018, the mean global AHEI was 40.3. The diet quality has increased from 1990 to 2018 in most parts of the world, but not in Sub-Saharan Africa (SSA). This clearly indicates the need of more focus on dietary issues in countries in SSA (1). Diet is an important modifiable risk factor associated with non-communicable diseases (2). Individual nutrients and foods, however, cannot be considered in isolation due to the complex interactions among nutrients (3, 4). Dietary pattern is an essential factor for the health of individuals and populations, which is also a key factor in the pattern of energy and nutrient intake (5). Changes in dietary patterns are not limited to the satisfaction of basal physiologic needs, but also are affected by social and cultural factors, including eating behavior (6). The discrepancy in increased energy intake and reduced expenditure results in energy imbalance, and when this is coupled with the reduction in physical activity becomes the underlying cause of overweight and obesity (7).

Dietary patterns that contain above the recommended quantity of energy-dense food items had an association with an increased burden of cardiovascular diseases (CVD) (2, 8). Globally, CVD remains the leading cause of mortality among middle-aged adults, and in high-income countries, it is the main cause of death next to cancer (8, 9). The dietary patterns in high-income countries are characterized by a high quantity of added sugars, fats, refined carbohydrates, and animal-source foods, which are termed a *western diet* (5, 8, 10).

Low- and middle-income countries (LMICs) are not immune to this problem. They are facing a double burden of diseases; and illnesses resulting from both under and over-nutrition (10). The incidence of non-communicable diseases such as cardiovascular diseases and type-2 diabetes is increasing due to changing lifestyles, urbanization, and increasing life expectancy (2, 11, 12). The dietary patterns in the LMICs, especially those with emerging economies are changing from the *traditional dietary pattern* with a high intake of fruits, vegetables, cereals, tubers, and legumes to *western diets* characterized by a high intake of energy-dense food items such as animal food products (13–15). The consumption of calories from meat, sugar, and vegetable oils increased significantly in developing countries between 1963 and 2003 (16).

Diets are determined by several factors, including individual and environmental factors. Urbanization independently or in combination with other factors is associated with changes in dietary patterns (17, 18). The urban food consumption pattern is generally more diversified; contains more animal products and sugar (7, 17, 18). A systematic review of data from forty countries in sub-Saharan Africa indicated variation in the dietary patterns between rural and urban areas (19). Factors including hypertension, blood cholesterol level, smoking, and physical activity were also related to dietary patterns (2, 5, 20, 21).

Ethiopia was frequently attacked by drought and famine during the past decades (22, 23). The total population living under the poverty line in 1994/95 was around 49.5%. However, after the application of various poverty reduction measures, the level of poverty in Ethiopia is decreasing (24). Succeeding economic growth in Ethiopia, the rate of urbanization is progressively increasing (24–27). Evidence indicates the coexistence of economic growth and urbanization results in lifestyle changes including changes in dietary patterns, which in turn may lead to increased obesity and nutrition-related NCDs diseases. This community-based study is the first of its kind in Ethiopia involving exploratory factor analysis to examine the dietary patterns among the adult population in the urban and rural areas of the study. Globally, the incidence of CVD is rising (2, 11, 12), and information on the association of cardiovascular risk factors with dietary patterns particularly in Ethiopia remains scant. Therefore, the current study aimed to assess the dietary patterns and study their association with cardiovascular risk factors among adult people in urban and rural areas of Wolaita, southern Ethiopia. This information might be useful for the promotion of healthy and traditional dietary patterns.

## Materials and methods

### Setting

The study was carried out in Wolaita, southern Ethiopia from May 2018 to February 2019. Wolaita has experienced rapid urbanization in the past 20 Years (28). We selected a town with the largest population size undergoing rapid urbanization, and a rural district with a relatively traditional lifestyle from Wolaita Zone.

Wolaita was one of the famine-affected and vulnerable areas in Ethiopia during 1983–1986 (22, 23). The livelihood of the urban population in Wolaita is based on employment, trade, or daily labor, while the livelihood of the rural population is based on crop production and animal husbandry (29). Access to food in rural areas depends on subsistence farming and is influenced by farm size, rainfall patterns, and crop production culture (29, 30).

### Study design, participants, and sampling technique

We conducted a community-based cross-sectional study. Two thousand four hundred eighty-three people aged 25–64 years participated in the study, and all invited to the study participated, except 3 people who were not available during three visits. The residents of randomly selected households from urban and rural areas were considered the study subjects. We selected the study participants by employing a three-stage survey. First, the survey *kebeles* (villages) were chosen randomly from a series of all registered *kebeles* in both study sites. Eleven out of 54 urban and ten out of 52 rural *kebeles* were included in our study. Secondly, we used a random integer generator to randomly choose households in the chosen *kebeles* from a list given to us by the community health workers (31). The list also had the names of the people living in each household. Thirdly, the number of study participants was decided proportionately to the size of their *kebeles* and households, and the participants were finally chosen from the eligible household members using a lottery method.

### Sample size

The sample size for this study was computed using Epi Info version 7 StatCalc software. This project is a part of a larger study, titled nutritional changes, and chronic diseases in Wolaita in southern Ethiopia (32, 33), and the number of participants was 2,486. We also considered assumptions from the study entitled prevalence of high blood pressure, hyperglycemia, dyslipidemia, metabolic syndrome and their determinants in Ethiopia: evidence from the national NCDs STEPS survey, 2015 to compute the sample size in one of the studies in our project (34). Accordingly, with a 14.9% prevalence of hypertension in rural, 19.7% prevalence of hypertension in urban, 95% confidence level, 80% power, one for the ratio of unexposed and exposed groups, and 10% non-response rate the total sample size became 2,233. In this study, since we aimed at assessing the dietary patterns, and their association with cardiovascular risk factors considering residence as the main exposure variable, we have computed posthoc power for the mean difference using OpenEpi version 3.03 software with 95% CI. Accordingly, the sample size to assess the dietary patterns (*western, traditional, and healthy*), and their association with cardiovascular risk factors taking residence as a primary exposure variable was adequate with the power of the study >90%.

### Data collection procedure and techniques

A total of 2,483 adult household members randomly selected for the survey were interviewed by trained data collectors, using a structured questionnaire about the socio-demographics such as age, education, wealth, diet, and other lifestyle factors. Additionally, we measured the anthropometric, blood pressure, and biomedical parameters of the study participants. The data collection process was undertaken within the participants’ homes, and people in the study obtained information about the data collection such as dates and overnight fasting from the supervisor and coordinator of the data collection before the data were collected. The blood samples were collected in the morning before eating breakfast.

The questionnaire was first designed in English and then translated into Amharic and Wolaitato. For validation, a re-translation was conducted by another expert. We provided training for the data collection team including nurses, laboratory technologists, field supervisor, coordinator, and data clerks for one week. The training consisted purpose of the survey, ethical conduct, and data collection techniques such as 24-h dietary recall assessment, interviewing skills, calibration of data collection instruments, and anthropometry. Following this, we conducted a pretest on 5% of our sample size among the population which was not selected for the survey. Subsequently, the inputs obtained from the pretest were incorporated into our data collection tool.

We adapted a quantitative 24-h dietary recall technique to serve as the data collection instrument for the previous day’s 24-h dietary intake assessment (35). To measure the dietary intakes at a population level, we employed single-day dietary histories on different individuals, the study population was selected randomly and all the days of the week were represented in the sample, and this was in line with the recommendation given by the principles of nutritional assessment (36). Since the previous day’s history is a recent memory, the interviewers asked the study participants to tell all the foods and beverages they consumed with their specific information such as preparation. We interviewed the participants by probing them recall all foods and beverages consumed during the previous day (from sunrise to sunrise) before the survey. The study participants were requested to provide specific information on foods and drinks including their product names and preparation techniques. We prepared a finite list of foods and beverages such as cereals, pulses, dairy products, vegetables, fruits, tubers, roots, meat and meat products, poultry, fish, egg, fats and oils, sugars, salt, coffee, and tea that helped to recall the previous day food and beverage consumption, and ticked off the mentioned items. In the end, the interview was finalized with the study participants confirming that all the foods and beverages they consumed during the previous day had been mentioned.

A participant’s physical activity was assessed by asking about activities during work, for instance carrying or lifting heavy loads. In addition, they were asked about their travel to and from places; e.g. walking and bicycling, and they were also asked about sports, fitness, and recreational activities (e.g., running, football, swimming). The activities were categorized as time spent on moderate-intensity and vigorous-intensity activities. The metabolic equivalent (MET) was calculated as each activity had a predefined value. MET-minutes/week of the specific activity was the product of the number of days in a week used to accomplish a given activity, the average time spent in minutes in a day, and the corresponding MET value. The overall MET-minutes/week was computed by summing the MET-minutes/week value of each activity (37, 38).

We also asked four questions about smoking habits (do you currently smoke any tobacco products; do you currently smoke tobacco products daily; do you currently use smokeless tobacco; do you currently use smokeless tobacco products daily).

A person’s BMI is computed by dividing weight in kg by height in m^2^. We quantified weight to the closest 0.1 kg by employing a mobile digital weighing scale (Seca electronic scale). We used a movable stadiometer comprising a suitable triangular headboard to quantify height (Seca stadiometer). The participants’ weight and height were measured while they stood straight, held their heads upright, and wore light clothing and shoes. During the height measurement, the external auditory of the ear and the bottom border of the eye were aligned in a single horizontal plane. In addition, the heels, shoulder blades, and buttocks touched the scale as the knees of the legs stayed together. Along with it, the arms were kept side by side. The participants’ heights were finally measured to the nearest 0.1 cm.

We measured blood pressure using a digital sphygmomanometer (Riester richampion^®^N, Germany). The participant’s blood pressure was taken three times following ten minutes of rest with the right upper arm positioned at the level of the heart. Systolic and diastolic blood pressures were determined by taking the average of the last two readings.

We took whole venous blood samples from each participant in the morning at their homes following an overnight fast. We collected blood samples in vacutainer tubes consisting of ethylenediaminetetraacetic acid, after cleaning the skin with a 70% alcohol swab. Then, the blood samples were kept in an icebox and transported to Wolaita Sodo University Hospital for analysis. Analysis of the blood samples was accomplished within twelve hours duration after acquisition. We used a BS-200 chemistry analyzer to investigate lipid profiles. Assessment of blood glucose (BG) was performed using a glucose meter (SensoCard^**®**^) at the site of blood sample collection.

### Operational definitions and categories for the analyses

The dietary patterns in our study were named after the food items or groups with the highest loadings in factor analysis, and related literature (2, 15, 39, 40). The *Western dietary pattern* consisted of meat/organ meat, biscuits/sweets, chicken stew, pasta-macaroni recipes, butter, white wheat bread, egg recipe, and *shiro-wet* food items or groups. *Shiro-wet* is an Ethiopian traditional dish mainly prepared using a mixture of the following ingredients: chickpea flour, red pepper flour, tomatoes, onions, garlic, oil, and sometimes butter. A *traditional dietary pattern* was characterized by the consumption of tubers, whole-grain maize products, coffee leaves-and-herbs beverage, legumes, and sweet potatoes. The food items or groups categorized under the *traditional dietary pattern* are culturally popular in the rural part of the study areas. In this study, green leafy vegetables, green peppers, and whole-grain maize products made up the *healthy dietary pattern* and were found in the urban area along with the food items or groups found in the *western dietary pattern*. Hyperglycemia was defined as having a blood glucose level ≥ 7.0 mmol/l, and/or self-reported use of medication for diabetes (41). A participant with total cholesterol (TC) level ≥ 5.2 mmol/L was categorized as having raised TC (42). A blood triglyceride (TG) level ≥ 1.7 mmol/l was defined as an elevated TG level (42). Hypertension was characterized by having a systolic blood pressure ≥ 140 mmHg, diastolic blood pressure ≥ 90 mmHg, and/or using medication for lowering the blood pressure (43). A body mass index (BMI) of 30 kg/m^2^ or greater indicates obesity (44). Having a level of physical activity <600 MET minutes per week was considered physically inactive (37, 38). Age was categorized into four using 10-year groups based on the WHO STEPS recommendation (37).

### Assessment of dietary patterns

Generally, dietary patterns are identified by using foods or nutrients, or a combination of both, and foods or food groups are often used as nutrients are composite food scores (45). Since the aim of this study was the identification of dietary patterns and associated cardiovascular factors, we used the previous day’s 24-h dietary intake to assess the dietary patterns. The dietary patterns were determined using factor analysis based on the intake of 24 food items or groups (45, 46). Some of the food items were categorized into groups depending on their similarities such as legumes, tubers, green leafy vegetables, and pasta and macaroni. Food items or groups with factor loadings ≥0.3 or ≤ −0.3 were considered as significantly contributing to the pattern. The number of factors that were retained in the analysis was determined based on the *eigenvalue* > 1.0, evaluation of the *scree plot*, and the plausibility of the factors. We used *orthogonal transformation* (*varimax rotation*) to identify uncorrelated factors and facilitate interpretability. Therefore, factor analysis and subsequent vari*max rotation* were used to determine the dietary patterns.

Positively loaded food items or groups contributed to a given dietary pattern, whereas negatively loaded foods have an opposite relation with a particular dietary pattern. A high factor score demonstrates a high intake of foods comprising a particular food pattern, whereas low scores demonstrate a low intake. Dietary patterns were named after the food items or groups with the highest loadings of those dietary patterns. Factor scores for each dietary pattern and participant were estimated by summing the consumption of each food item or group weighted by their factor loadings. Subsequently, the *tertiles* of the dietary patterns scores were generated by classifying the scores into three categories: first tertile (lowest), second tertile, and third tertile (highest) to show the frequencies in relation to other variables in the descriptive tables. The association of dietary patterns with CVD risk factors was analyzed using bivariate and multiple linear regressions, and cross-tabulation was used for descriptive analysis.

### Data entry and analysis

Data entry was accomplished using Epi-Data version 3.1 and excel-template, and exported to the STATA 15 software for analysis. We have performed residence-specific principal component analysis to build the wealth index, using 40 variables for rural and 28 variables for urban areas. Detailed information on wealth index construction was reported in a previous publication from the project, and in this study, it was categorized as poor, medium, and rich (33).

The prevalence and frequencies of tertiles of dietary patterns were calculated. The outcome variables were dietary patterns that were identified using factor analysis. The covariates used in this analysis include residence, education, physical activity, obesity, hypertension, hyperglycemia, total cholesterol, and triglyceride levels. The associations between covariates and dietary patterns were assessed using bivariable and multivariable linear regression models building a separate model for each of the identified dietary patterns. The data analysis was started after declaring the data set as a three-stage cluster survey to account for the effect of clustering on the estimated standard errors. The assumption of normality of the continuous variables was checked objectively using *sktest* (skewness-kurtosis test) and subjectively using histograms. Based upon this the natural logarithmic transformation was made for the outcome variables to satisfy the assumption. The result demonstrated the normal distribution of the residuals. Variables with the *p* values <0.2 in the bivariable analysis were considered a candidate for multiple linear regression analysis. The adjusted regression coefficient with its 95% CI is presented, and the absence of 0 within the 95% CI declared the presence of association.

### Ethical considerations

This study was approved by both the Institutional Review Board at Hawassa University in Ethiopia (IRB/005/10) and the Regional Committee for Medical Research Ethics Northern Norway, REK North (2017/2248/REK nord). The study subjects provided written informed consent following the introduction of the purpose of the study. Individuals in the study remained anonymous, except those having hyperglycemia, hypertension, or other serious ailments that were referred to the closest health facility.

## Results

### Socio-demographic characteristics of the participants

A total of 2,483 adults participated in the study of 2,486 invited people. The number of male people involved in the study was 1,313 (52.9%). There was a relatively equal level of participation between urban (50.1%) and rural (49.9%) study areas. Of the total study participants 1,085 (43.7%), 674 (27.1%), 441 (17.8%), and 283 (11.1%) were aged between 25–34, 35–44, 45–54, and 55–64 years, respectively. Concerning the educational level of the study participants: 1410 (56.8%) had a primary level of education or below, 397 (16.0%) had high school, and 676 (27.2%) had college or education above this level. Calculating the wealth index analysis, we have found 784 (31.6%) poor participants, 793 (31.9%) medium level, and 906 (36.5%) rich.

Of the total study participants, 47.2% (1172) had a physical activity status of ≥600 MET minutes per week. One hundred nine (4.4%) of the overall study participants were obese. The prevalence of hypertension was 32.9% (818), while hyperglycemia was 4.4% (110). Hypercholesterolemia was detected in 5.7% (142), and hypertriglyceridemia in 15.8% (393) of the study participants. Eighteen (0.7%) people were reported to be daily smokers.

### Dietary patterns

Three dietary patterns were distinguished, describing 51% of the total variance in food consumption using factor analysis. The dietary patterns with their rotated factor loadings are illustrated in Table 1. The first pattern comprised animal-source foods with added sugars and refined carbohydrates that resemble the *western type dietary pattern*. This pattern explained 21.5% of the total variance in food intake. Food items or groups with the highest factor loadings such as meat/organ meat, and biscuits/sweets positively contributed to the *western dietary pattern*, whereas whole grain maize products had a negative contribution. The second pattern demonstrated a high intake of plant-based food sources that matches a *traditional dietary pattern* explaining 18.1% of the total variance. This pattern consists of tubers having the highest positive factor loading. The third pattern was termed the *healthy dietary pattern* which explained 11.4% of the total variance and featured the consumption of green vegetables and whole-grain maize products (See Table 1).

**Table 1:** Dietary patterns found in factor analysis, with their rotated factor loadings in an Ethiopian population (*n* = 2,483).

Food items/groups	Dietary patterns			*H* ^2^
	Western	Traditional	Healthy	
Meat/ organ meat	0.8014	−0.0827	−0.1815	0.3179
Biscuits/ sweets	0.7479	0.0347	0.0086	0.4393
Chicken stew	0.7287	0.0084	0.0220	0.4684
Pasta/ macaroni recipe	0.6327	−0.2366	−0.1959	0.5054
Butter	0.6235	−0.4004	−0.3489	0.3292
White wheat bread	0.6106	−0.3523	−0.2858	0.4214
Egg/ egg recipe	0.5742	−0.0298	−0.0103	0.6693
Shiro-wet	0.4199	−0.6664	−0.0985	0.3699
Whole grain maize products	−0.4290	0.5735	0.3546	0.3613
Coffee beverage	0.1005	−0.1015	−0.5522	0.6747
Green leafy vegetables	−0.1956	−0.0890	0.7690	0.3625
Green pepper	−0.1115	0.0885	0.6705	0.5302
Tubers	−0.1021	0.7064	0.0648	0.4864
Legumes	−0.0290	0.5340	0.2611	0.6458
Sweet potato	−0.0084	0.5028	0.0635	0.7431
Teff injera	−0.0063	−0.7722	0.2832	0.3234
Coffee leaves-herb beverage	−0.0003	0.5509	0.1407	0.6767

H^2^: Communality; Shiro-wet: Ethiopian dish mainly prepared using a mixture of the following ingredients: chickpea flour, red pepper flour, tomatoes, onions, garlic, oil, and sometimes butter.

### Description of dietary patterns scores and CVD risk factors

Participants with the highest tertile of the *western and healthy dietary patterns* tended to reside in the urban study area, while those with the highest tertile of *traditional dietary pattern* resided in the rural area. We observed a higher level of physical activity among participants with the highest tertiles of the *traditional dietary pattern* and those who lived in rural areas. A higher occurrence of obesity was observed among participants with the upper tertile of the *western* and the second but not the third tertile of the *traditional dietary pattern*, while obesity was lower among participants with the upper tertile of both *traditional and healthy dietary patterns*. Hypertension increased among the adult people with the highest tertiles of the *western dietary pattern* and decreased among people with the highest tertiles of the *traditional and healthy dietary patterns*. Similarly, people in the highest tertiles of the *western dietary pattern* had an increased level of hyperglycemia and elevated total cholesterol levels. We found no difference in smoking rates across the increasing tertiles of dietary patterns (Table 2).

**Table 2:** Socio-demographic, behavioral, and biochemical characteristics across tertiles of the three dietary patterns scores identified among adults in Wolaita, southern Ethiopia.

Variables (*n* = 2,483)	Dietary patterns								
	Western			Traditional			Healthy		
	T1	T2	T3	T1	T2	T3	T1	T2	T3
*Age (year)*	*n* (%)	*n* (%)	*n* (%)	*n* (%)	*n* (%)	*n* (%)	*n* (%)	*n* (%)	*n* (%)
25–34	365 (33.6)	404 (37.2)	316 (29.1)	372 (34.3)	321 (29.6)	392 (36.1)	310 (28.6)	360 (33.2)	415 (38.3)
35–44	239 (35.5)	219 (32.5)	216 (32.1)	184 (27.3)	202 (30.0)	288 (42.7)	229 (34.0)	240 (35.6)	205 (30.4)
45–54	125 (28.3)	118 (26.8)	198 (44.9)	133 (30.2)	150 (34.0)	158 (35.8)	175 (39.7)	141 (32.0)	125 (28.3)
55–64	95 (33.6)	82 (29.0)	106 (37.5)	76 (26.9)	103 (36.4)	104 (36.8)	112 (39.6)	85 (30.0)	86 (30.4)
*Gender*									
Female	366 (31.3)	386 (33.0)	418 (35.7)	390 (33.3)	358 (30.6)	422 (36.1)	364 (31.1)	403 (34.4)	403 (34.4)
Male	458 (34.9)	437 (33.3)	418 (31.8)	375 (28.6)	418 (31.8)	520 (39.6)	462 (35.2)	423 (32.2)	428 (32.6)
Residence									
Rural	560 (45.2)	393 (31.7)	287 (23.2)	26 (2.1)	312 (25.2)	902 (72.7)	445 (35.9)	462 (37.3)	333 (26.9)
Urban	264 (21.2)	430 (34.6)	549 (44.2)	739 (59.5)	464 (37.3)	40 (3.2)	381 (30.7)	364 (29.3)	498 (40.1)
*Education*									
≤ Primary	545 (38.7)	461 (32.7)	404 (28.7)	225 (16.0)	414 (29.4)	771 (54.7)	479 (34.0)	478 (33.9)	453 (32.1)
High school	116 (29.2)	141 (35.5)	140 (35.3)	139 (35.0)	147 (37.0)	111 (28.0)	119 (30.0)	140 (35.3)	138 (34.8)
College+	163 (24.1)	221 (32.7)	292 (43.2)	401 (59.3)	215 (31.8)	60 (8.9)	228 (33.7)	208 (30.8)	240 (35.5)
*Wealth index*									
Poor	273 (34.8)	258 (32.9)	253 (32.3)	235 (30.0)	258 (32.9)	291 (37.1)	281 (35.8)	252 (32.1)	251 (32.0)
Medium	263 (33.2)	248 (31.3)	282 (35.6)	222 (28.0)	251 (31.7)	320 (40.4)	272 (34.3)	245 (30.9)	276 (34.8)
Rich	288 (31.8)	317 (35.0)	301 (33.2)	308 (34.0)	267 (29.5)	331 (36.5)	273 (30.1)	329 (36.3)	304 (33.6)
*Physical activity*									
No	436 (33.3)	393 (30.0)	482 (36.8)	573 (43.7)	476 (36.3)	262 (20.0)	490 (37.4)	385 (29.4)	436 (33.3)
Yes	388 (33.1)	430 (36.7)	354 (30.2)	192 (16.4)	300 (25.6)	680 (58.0)	336 (28.7)	441 (37.6)	395 (33.7)
*Smoking*									
No	817 (33.1)	818 (33.2)	830 (33.7)	764 (31.0)	767 (31.1)	934 (37.9)	820 (33.3)	818 (33.2)	827 (33.6)
Yes	7 (38.9)	5 (27.8)	6 (33.3)	1 (5.6)	9 (50.0)	8 (44.4)	6 (33.3)	8 (44.4)	4 (22.2)
*Obesity*									
No	824 (37.4)	816 (34.4)	734 (30.9)	737 (31.0)	720 (30.3)	917 (38.6)	778 (32.8)	772 (32.5)	824 (34.7)
Yes	0 (0)	7 (6.4)	102 (93.4)	28 (25.7)	56 (51.4)	25 (22.9)	48 (44.0)	54 (49.5)	7 (6.4)
*Hypertension*									
No	548 (32.9)	697 (41.9)	420 (25.2)	498 (29.9)	403 (24.2)	764 (45.9)	301 (18.1)	576 (34.6)	788 (47.3)
Yes	276 (33.7)	126 (15.4)	416 (50.9)	267 (32.6)	373 (45.6)	178 (21.8)	525 (64.2)	250 (30.6)	43 (5.3)
*Hyperglycemia*									
No	795 (33.5)	819 (34.5)	759 (32.0)	743 (31.3)	709 (29.9)	921 (38.8)	783 (33.0)	773 (32.6)	817 (34.4)
Yes	29 (26.4)	4 (3.6)	77 (70.0)	22 (20.0)	67 (60.9)	21 (19.1)	43 (39.10)	53 (48.2)	14 (12.7)
*TC*									
Low	794 (33.9)	794 (33.9)	753 (32.2)	723 (30.9)	700 (29.9)	918 (39.2)	748 (32.0)	783 (33.5)	810 (34.6)
High	30 (21.1)	29 (20.4)	83 (58.5)	42 (29.6)	76 (53.5)	24 (16.9)	78 (54.9)	43 (30.3)	21 (14.8)
*TG*									
Low	699 (33.4)	725 (35.0)	666 (31.9)	651 (31.2)	605 (29.0)	834 (39.9)	651 (31.2)	685 (32.8)	754 (36.1)
High	125 (31.8)	98 (24.9)	170 (43.3)	114 (29.0)	171 (43.5)	108 (27.5)	175 (44.5)	141 (35.9)	77 (19.6)

T1: Tertile 1; T2: Tertile 2; T3: Tertile 3.

### Cardiovascular factors and association with dietary patterns

**Figure 1:**
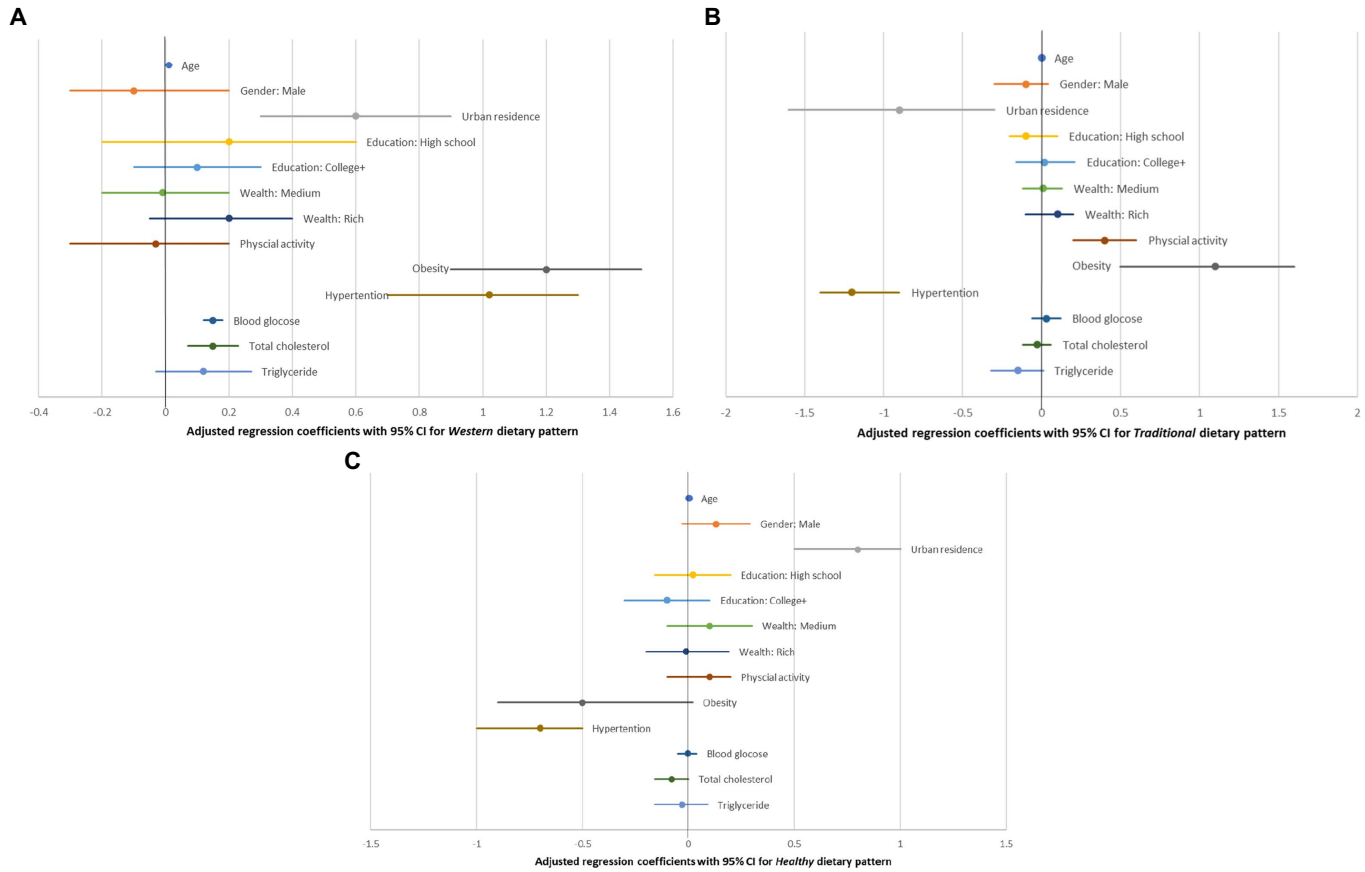
**(A–C)** Show the adjusted regression coefficients in relation to their position to the null value of zero for each of the three dietary patterns. The numerical values of the coefficients along with their crude estimates were depicted in Table 3.

The study participants having obesity were positively associated with western [*β* = 1.2; 95% CI: 0.9–1.5], and traditional [*β* = 1.1; 95% CI: 0.5–1.6] dietary patterns; while they were inversely associated with the *healthy dietary pattern* [*β* = −0.48; 95% CI: −0.94, −0.02]. Similarly, participants who developed hypertension were positively associated with the *western dietary pattern* [*β* = 1.0; 95% CI: 0.7–1.3], as they were negatively associated with the *traditional* [*β* = −1.2; 95% CI: −1.4, −0.9], *and healthy* [*β* = −0.7; 95% CI: −0.9, −0.4] dietary patterns. Being a resident in the urban area was positively related to the *western dietary pattern* [*β* = 0.6; 95% CI: 0.3–0.8]. Urban residence was also positively associated with the *healthy dietary pattern* [*β* = 0.8; 95% CI: 0.5–1.0]. Meanwhile, urban residence had an inverse relationship with the *traditional dietary pattern* [*β* = −0.9; 95% CI: −1.6, −0.2]. We found a positive linear association between the *traditional dietary pattern* and physical activity [*β* = 0.4; 95% CI: 0.2–0.6]. Moreover, there was an increasing linear association between blood glucose levels and the *western dietary pattern* [*β* = 0.15; 95% CI: 0.11–0.18]. We also observed an increased *western dietary pattern* with the increasing total cholesterol level [*β* = 0.15; 95% CI: 0.07–0.23], after adjusting for the other factors in the model (See Table 3; Figures 1A–C).

**Table 3:** Multivariable log-linear regression analysis of socio-demographic, behavioral, and biochemical factors with dietary patterns among adults in Wolaita, southern Ethiopia.

Variables (*n* = 2,483)	Dietary patterns					
	Western		Traditional		Healthy	
	Crude β (95% CI)	Adjusted β (95% CI)	Crude β (95% CI)	Adjusted β (95% CI)	Crude β (95% CI)	Adjusted β (95% CI)
Age (year)	0.03 (0.01, 0.04)	0.01 (−0.001, 0.02)	0.007 (−0.013, 0.001)	0.002 (−0.003, 0.01)	0.002 (−0.005, 0.01)	0.004 (−0.001, 0.01)
Gender						
Female	0	0	0	0	0	0
Male	−0.1 (−0.4, 0.2)	−0.1 (−0.3, 0.2)	−0.07 (−0.26, 0.12)	−0.1 (−0.3, 0.04)	0.15 (−0.02, 0.32)	0.13 (−0.03, 0.29)
Residence						
Rural	0	0	0	0	0	0
Urban	1.6 (1.4, 1.8)	0.6 (0.3, 0.9)	−1.31 (−1.58, −1.03)	−0.9 (−1.6, −0.3)	0.59 (0.42, 0.75)	0.8 (0.5, 1.0)
Education						
≤ Primary	0	0	0	0	0	0
High school	0.7 (0.4, 1.1)	0.2 (−0.2, 0.6)	−0.23 (−0.51, 0.06)	−0.1 (−0.2, 0.1)	0.24 (0.09, 0.39)	0.02 (−0.16, 0.20)
College+	0.8 (0.4, 1.2)	0.1 (−0.1, 0.3)	−0.65 (−1.13, −0.16)	0.02 (−0.16, 0.21)	0.30 (0.05, 0.55)	−0.1 (−0.3, 0.1)
Wealth index						
Poor	0	0	0	0	0	0
Medium	0. 1 (−0.2, 0.4)	−0. 01 (−0.2, 0.2)	0.03 (−0.16, 0.21)	0.01 (−0.12, 0.13)	0.05 (−0.15, 0.25)	0.1 (−0.1, 0.3)
Rich	0.3 (−0.1, 0.7)	0.2 (−0.05, 0.4)	0.04 (−0.16, 0.24)	0.1 (−0.1, 0.2)	−0.0004 (−0.24, 0.23)	−0.01 (−0.20, 0.19)
Physical activity						
No	0	0	0	0	0	0
Yes	−0.8 (−1.1, −0.4)	−0.03 (−0.3, 0.2)	0.6 (0.3, 0.8)	0.4 (0.2, 0.6)	−0.24 (−0.35, −0.14)	0.1 (−0.1, 0.2)
Obesity						
No	0	0	0	0	0	0
Yes	1.5 (1.2, 1.9)	1.2 (0.9, 1.5)	−0.6 (−0.9, −0.4)	1.1 (0.5, 1.6)	−0.54 (−0.82, −0.25)	−0.5 (−0.9, −0.02)
Hypertension						
No	0	0	0	0	0	0
Yes	1.4 (1.1, 1.8)	1.02 (0.7, 1.3)	−1.3 (−1.5, −1.2)	−1.2 (−1.4, −0.9)	−0.58 (−0.84, −0.32)	−0.7 (−1.0, −0.5)
BG (mmol/L)	0.23 (0.19, 0.26)	0.15 (0.12, 0.18)	−0.07 (−0.11, −0.04)	0.03 (−0.06, 0.12)	−0.04 (−0.08, 0.002)	−0.003 (−0.05, 0.04)
TC (mmol/L)	0.3 (0.2, 0.5)	0.15 (0.07, 0.23)	−0.22 (−0.31, 0.12)	−0.03 (−0.12, 0.06)	−0.04 (−0.12, 0.04)	−0.08 (−0.16, 0.001)
TG (mmol/L)	0.5 (0.2, 0.7)	0.12 (−0.03, 0.27)	−0.34 (−0.50, −0.18)	−0.15 (−0.32, 0.01)	−0.07 (−0.19, 0.06)	−0.03 (−0.16, 0.09)

β, Beta coefficient; BG, Blood glucose; TC, Total cholesterol; TG, Triglyceride.

As only 18 people were smoking, this variable was not included in the main analyses shown in Table 3. However, an analysis including this variable was performed, but this did not influence the results (data not shown).

## Discussion

This community-based study is the first of its kind in Ethiopia involving exploratory factor analysis to examine the dietary patterns among the adult population. The study mainly identified the intake of three dietary patterns. The first *dietary pattern* was *western* which is characterized by the consumption of meat/organ meat, biscuits/sweets, chicken stew, pasta-macaroni recipes, butter, white wheat bread, egg recipe, and *shiro-wet.* Urban residence, obesity, hypertension, blood glucose, and total cholesterol levels were positively associated with the *western dietary pattern*. Adherence to the consumption of tubers, whole-grain maize products, coffee leaves-and-herbs beverage, legumes, and sweet potatoes featured the *traditional dietary pattern*. It showed a positive relationship with rural residence, physical activity, and obesity, while it had a negative relationship with hypertension. The *healthy dietary pattern* was characterized by the intake of green leafy vegetables, green pepper, and whole-grain maize products, and negatively related to urban residence, obesity, and hypertension.

In this study, we found a more significant association of the *western, and healthy dietary patterns* with the urban part of the population, compared to the rural population after controlling for potential confounders. The observed relationship between the *western dietary pattern* and the urban environment is in agreement with the findings reported elsewhere in the LMICs (20, 39, 47). This might be due to lifestyle changes related to the rapidly growing urbanization in Wolaita (20, 48). However, this study also indicated the presence of a *healthy dietary pattern* in the urban environment. This might indicate the emergence of transition to new diets in the study area. Furthermore, some individuals might have the awareness of the importance of healthy dietary choices. In contrast, we recorded a significant relationship between the *traditional dietary pattern* and the rural populations. This is consistent with the findings of other studies (20, 49). This might be the reason that traditional dishes are commonly consumed by the rural population. Further, adherence to the *traditional dietary pattern* was associated with physical activity. This is consistent with the finding reported elsewhere in West Africa (49). In rural areas, moderate or vigorous activities like farming may account for the observed relationship.

Consumption of *western and traditional dietary patterns* was associated with obesity. The finding regarding the relationship between *western dietary pattern* and obesity was supported by various studies (2, 13, 47). Compliance with the western diet is associated with higher energy intake, which accounts for weight gain and increased risk of obesity (50, 51). With a further look at the positive association between the *traditional dietary pattern* and obesity, we noted that the proportion of obesity was not higher for the third tertile (23%), but it was for the second tertile (51%), both compared to the first tertile (26%) showing this association was not straight forward. This might also be due to the limitation of a single 24-h dietary intake assessment not representing a long-term dietary habit, even though population-level usual dietary intake can be measured using a single-day dietary assessment provided that the study participants were selected randomly, and all days of the week are represented in the sample (36). But there exists some evidence from Asian countries that indicate a positive relationship between *traditional dietary pattern* and obesity (52–54). However, we suggest further investigation to ascertain this relationship in the context of the study area. Meanwhile, a *healthy dietary pattern* appears to be inversely associated with obesity, which is supported by the findings of other studies (13, 55).

Hypertension was positively associated with the *western dietary pattern* (2), as it was negatively associated with the *traditional and healthy dietary patterns* (21, 56). There has been little understanding of the mechanisms involved. However, the mechanisms linked to the etiology of arterial hypertension brought on by the western diet are complex, and include several factors. High salt intake is one of the best-known risk factors for hypertension (57), but a number of different antioxidants are also associated with the development of hypertension (58). There was a positive linear association between blood glucose levels and a *western dietary pattern*. Similar findings were reported elsewhere (2, 15, 52). As a pro-inflammatory diet, the western diet can trigger inflammatory markers and cytokines and increase oxidative stress, which in combination lead to cell and DNA damage, reducing insulin receptors, and lowering insulin production (59). Similarly, total cholesterol levels increased with adherence to the *western dietary pattern*. Increases in plasma cholesterol may occur if the sources of cholesterol are consumed along with saturated and trans fats, as is the case with the *western dietary pattern* (60). Other community-based studies have also revealed similar results (2, 61, 62). Unlike *Western dietary pattern*, cardio-metabolic risk factors such as total cholesterol, triglyceride, and blood glucose levels were not associated with *traditional and healthy dietary patterns*. This is supported by the findings from other studies (63–66). The mechanisms by which *traditional and healthy dietary patterns* are linked to cardio-metabolic risk factors are not fully understood. However, the absence of association may be due to the high fiber and low glycemic load of plant-based food items such as whole grains, legumes, and vegetables in *traditional and healthy dietary patterns* (67). The population had very few smokers, and this factor is very unlikely to have influenced the results. This is not the situation in most other studies, and this makes the present study quite unique.

The findings of this study may have public health significance through promotion of *healthy and traditional dietary patterns* along with physical activity. Interventions related to the findings, if initiated early in life, may benefit the public in preventing cardiovascular risk factors such as obesity, hypertension, and type-2 diabetes (68–72). Furthermore, this study has policy implications in terms of the importance of focusing on nutrition-related non-communicable diseases and provides latest data on dietary patterns for policies related to nutrition.

### Strengths and limitations

The design of the present study was cross-sectional. A cross-sectional study with a 24-h dietary intake assessment is a single-day experience and does not guarantee the understanding of the usual dietary pattern, and lacks temporal relations. The causality between the diets and the risk factors cannot be interpreted, but still, findings from this study bring forward new information that might be useful in the understanding of diet and other factors.

The response rate in this study was very high, as 2,483 people participated, out of a population of 2,486. The reason for the high response is likely to be the provision of information including the objective of the study and schedules before the data collection, and repeated visits to the homes in their absence.

Interviews were used for obtaining information from the participants. This is a feasible method in a population where some individuals might be illiterate and others are not used to writing at all. Also, many do not have much knowledge about nutrition and health (73).

Information was obtained for all interviewed individuals using a 24-h recall method and this has been used in low-income settings for many years. The method has been debated whether it can serve as a substitute for the weighted food records to assess the absolute nutrient intake (74, 75). Nevertheless, in this study, the amount of food and type of nutrient intake was not required as we aimed at identifying the dietary patterns.

It is very difficult to register food consumption in a population. Recall bias might be present, and the answers received in the present study might not be accurate. Another method that could have been used is weighted records. However, this requires more resources, and may also have uncertainties due to the workload put on the people who must weigh their food and record it.

Smoking is one of the major modifiable CVD risk factors. However, in our study, we did not investigate the relationship between smoking and dietary patterns because of the smaller number of smoking participants.

There might be misclassifications in our study related to the blood glucose and lipid profile. Although the study participants were told to have been fasting before the blood test, we cannot be confident that this was the situation for everyone. We tried to reduce this weakness of the study by giving the participants the required information before the examination day.

The study had several research assistants and which may increase inter-observer bias, and to minimize this, all were trained together and a common understanding of the tools was assured as much as possible.

The findings of this study might be valid for Ethiopian populations. However, dietary issues might not be similar in other cultures and countries, and the results may not be generalized outside of the country.

For even more certain conclusions, future studies should have a longitudinal design with repeated measurements of the diet. It would also be of interest to study the presence of cardiovascular diseases in a longitudinal setting, but this must be done with caution due to the ethical considerations needed.

### Conclusion

The coexistence of western, *traditional, and healthy dietary patterns* in the present study may indicate the transition to a new dietary pattern among people in the study area. All dietary patterns were associated with one or more cardiovascular risk factors, but the *western dietary pattern* was associated with most of these. The *traditional diet* showed fewer such associations. Therefore, it might be useful to promote *healthy and traditional dietary patterns* along with physical activity. Interventions related to the current findings, if initiated early in life, may benefit the public in preventing cardiovascular risk factors such as obesity, hypertension, and type 2-diabetes. Furthermore, this study may have policy implications in terms of the importance of focusing on nutrition-related non-communicable diseases, and provides recent data on dietary patterns for policies related to nutrition. Hence, this information should be disseminated and discussed both at local and national levels in Ethiopia.

## Data availability statement

The raw data supporting the conclusions of this article will be made available by the authors, without undue reservation.

## Ethics statement

The studies involving human participants were reviewed and approved by both the Institutional Review Board at Hawassa University in Ethiopia (IRB/005/10) and the Regional Committee for Medical Research Ethics Northern Norway, REK North (2017/2248/REK nord). The patients/participants provided their written informed consent to participate in this study.

## Author contributions

WK and EL conceptualized and planned the study, performed the formal analysis of the data, review, editorial activities, and approved the final version of the manuscript. WK carried the protocol development, data collection, supervision activities, and prepared the original manuscript. All authors contributed to the article and approved the submitted version.

## Funding

This study was granted by the Norwegian Program for Capacity Development in Higher Education and Research for Development. Award/grant number: ETH-13-0025.

## Conflict of interest

The authors declare that the research was conducted in the absence of any commercial or financial relationships that could be construed as a potential conflict of interest.

## Publisher’s note

All claims expressed in this article are solely those of the authors and do not necessarily represent those of their affiliated organizations, or those of the publisher, the editors and the reviewers. Any product that may be evaluated in this article, or claim that may be made by its manufacturer, is not guaranteed or endorsed by the publisher.
